# The role of cullin 5-containing ubiquitin ligases

**DOI:** 10.1186/s13008-016-0016-3

**Published:** 2016-03-09

**Authors:** Fumihiko Okumura, Akiko Joo-Okumura, Kunio Nakatsukasa, Takumi Kamura

**Affiliations:** Division of Biological Science, Graduate School of Science, Nagoya University, Nagoya, Aichi 464-8602 Japan

**Keywords:** Ubiquitin, Cullin 5, Elongin, CRL complex

## Abstract

The suppressor of cytokine signaling (SOCS) box consists of the BC box and the cullin 5 (Cul5) box, which interact with Elongin BC and Cul5, respectively. SOCS box-containing proteins have ubiquitin ligase activity mediated by the formation of a complex with the scaffold protein Cul5 and the RING domain protein Rbx2, and are thereby members of the cullin RING ligase superfamily. Cul5-type ubiquitin ligases have a variety of substrates that are targeted for polyubiquitination and proteasomal degradation. Here, we review the current knowledge on the identification of Cul5 and the regulation of its expression, as well as the signaling pathways regulated by Cul5 and how viruses highjack the Cul5 system to overcome antiviral responses.

## Identification and regulation of cullin 5

Cullin 5 (Cul5) was originally identified as a vasopressin-activated calcium-mobilizing (VACM-1) protein, an arginine vasopressin (AVP) receptor [1]. AVP is a nonapeptide that regulates body fluid and blood pressure homeostasis. VACM-1 is recognized as Cul5 because of its homology to the *Caenorhabditis elegans* gene *Cul5* [2, 3]. Cul5 is expressed in many cells and organs, including endothelial cells, brain, kidney collecting tubule cells, and vascular endothelial cells [2, 4–6, 7]. Cul5 inhibits cyclic AMP production, and this effect is reversed by staurosporin, a protein kinase A (PKA) inhibitor, or by mutating S730A, the PKA-dependent phosphorylation site in the Cul5 sequence in COS-1 cells [8]. The inhibitory effect of Cul5 on AVP-stimulated cAMP production is enhanced by a protein kinase C inhibitor [8]. *CUL*-*5* expression is downregulated in 82 % (41/50) of breast tumors compared with matched normal tissues [9]. Overexpression of Cul5 in T47D breast cancer cells decreases cell growth and mitogen activated protein kinase (MAPK) phosphorylation [10], and Cul5 overexpression downregulates early growth response 1 (EGR-1) protein expression and upregulates Fas-L mRNA expression [10]. The regulation of both MAPK and EGR-1 pathways by 17β-estradiol led to the examination of estrogen-dependent T47D cell growth, which showed that Cul5 inhibits basal and 17β-estradiol-dependent cell growth and MAPK phosphorylation [11].

Resveratrol (trans-3,5,4′-trihydroxystilbene), which inhibits tumor initiation and promotion, is a natural component of the human diet, and its wide range of biological activities has been demonstrated in vivo and in vitro [12–15]. The antiproliferative effect of resveratrol is significantly enhanced by Cul5 overexpression in T47D cells [16].

**Fig. 1:**
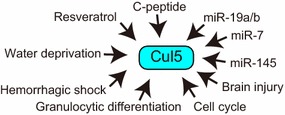
Regulation of Cul5. Several stimuli or microRNAs regulate the expression of Cul5

The expression of Cul5 is regulated by several stimuli and pathways (Fig. 1). Resveratrol upregulates Cul5 expression and decreases T47D cell growth, suggesting that the antiproliferative effect of resveratrol is mediated by Cul5 [16]. Cul5 is a flexible scaffold protein with a preferred distribution of conformational states [17], and NEDD8 modification (neddylation) alters the conformation of Cul5 and activates it [18]. Cul5(S730A) accelerates cellular proliferation and induces angiogenic growth in rat adrenal medullary endothelial cells (RAMECs) [19]. Cul5 neddylation is increased by the S730A mutation, and activation of PKA by forskolin suppresses the neddylation of Cul5 [20]. Furthermore, PKC-induced RAMEC proliferation is enhanced by Cul5(S730A) [20]. Cul5(S730A) expression in RAMECs increases the levels of phosphorylated MAPK and the translocation of the transcription factor EGR-1, a tumor suppressor, to the nucleus; it also causes morphological alterations mediated by actin rearrangement [19]. Furthermore, Cul5(S730A) downregulates maspin, a putative tumor suppressor [21] that is essential for early embryonic development [22], although these functions are controversial [23]. These reports suggest that Cul5 plays a role in endothelial cell growth and angiogenesis by regulating MAPK phosphorylation, the nuclear localization of EGR-1, maspin expression, and actin polymerization. Nevertheless, no mutation was found at the putative phosphorylation or neddylation site of Cul5 in T47D breast cancer cells, U138MG glioma cells, ACHN renal cancer cells, and OVCAR-3 ovarian cancer cells [24]. *C. elegans* oocyte septum formation and egg production were absent in Cul5- or ring box protein 2 (Rbx2)-depleted Cul2 homozygotes, whereas control Cul2 homozygotes laid approximately 50 eggs [25]. Additionally, Cul5-depleted Cul2 mutants and Cul2-depleted Cul5 mutants show decreased MPK-1 activity, suggesting that oocyte maturation from pachytene exit and MPK-1 activation are redundantly controlled by the Rbx2-Cul5- and Rbx1-Cul2-based complexes [25].

C-peptide [26, 27], the product of the cleavage of proinsulin, is a peptide hormone that acts through a G protein-coupled membrane receptor [28–30]. Given that C-peptide and vasopressin share similar intracellular effects, including the activation of calcium influx and endothelial nitric oxide (NO) synthase [31–36], the effect of C-peptide on Cul5 was examined [37]. Cul5 expression was increased by C-peptide, and the induction was prevented by pertussis toxin, a specific inhibitor of G proteins [37].

Rat *Cul5* mRNA is expressed in the brain and its levels increase in the rat cerebral cortex, hypothalamus, and kidney in response to 48 h of water deprivation [38, 39]. Cul5 overexpression in COS-1 cells downregulated aquaporin-1 (AQP1), and Cul5 was upregulated in rat mesenteric arteries, skeletal muscle, and the heart ventricle in response to 24 h of water deprivation [40]. Cul5 neddylation was also increased by 24 h of water deprivation, and AQP1 levels were inversely correlated with the ratio of Cul5 to neddylated Cul5 [40]. Furthermore, overexpression of Cul5 downregulated AQP2, and Cul5 was decreased in renal collecting ducts in response to water deprivation [41]. Cul5 mRNA levels were increased in the brainstem and cerebellum, and decreased in the hypothalamus of rats by hemorrhagic shock [42].

Cul5 disappears during the cell cycle S phase; it localizes to the cytosol during cell division and to the cell membrane at the completion of cytokinesis, suggesting that it plays a role in cell division [43]. Cul5 mRNA and protein levels are decreased in the rat cerebral cortex and hippocampus in response to traumatic brain injury (TBI) [44]. Another report showed a 6.5-fold upregulation of Cul5 associated with granulocytic differentiation of HL-60 cells [45].

Hepatitis B virus infection downregulates microRNA-145 (miR-145), upregulates Cul5 expression, and enhances cell proliferation [46]. miR-7, which upregulates Cul5 expression, is downregulated in hepatocellular carcinoma (HCC) tissues compared with adjacent non-tumor tissue [47]. By contrast, overexpression of miR-7 prevents colony formation and induces G1/S phase arrest, suggesting that miR-7 is a tumor suppressor in HCC [47]. miR-19a and -19b (miR-19a/b), which negatively regulate Cul5 expression, are highly expressed in human cervical cancer cells [48]. Upregulation of miR-19a/b promotes cell growth and invasion, whereas overexpression of miR-19a/b-resistant Cul5 without its 3′-UTR abolishes the effect of miR-19a/b on cell proliferation and invasion [48].

Rbx2 is polyubiquitinated by NEDD4-1, a HECT domain-containing E3 ubiquitin ligase, and targeted for proteasome-mediated degradation, suggesting that NEDD4-1 suppresses Cul5 ubiquitin ligase activity [49]. Overexpression of NEDD4-1 increases etoposide-induced apoptosis, suggesting that Rbx2 has an anti-apoptotic role [49, 50].

## Cul5-containing ubiquitin ligases

### CIS/SOCS family

**Fig. 2:**
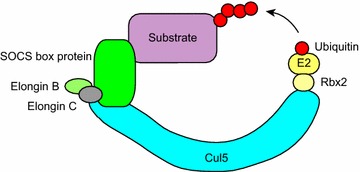
Cul5-containing ubiquitin ligases. Cul5 is a scaffold protein that recruits Rbx2, the Elongin B/C complex, and SOCS box proteins. SOCS box proteins recognize particular substrates to be polyubiquitinated

Suppressor of cytokine signaling (SOCS) proteins (SOCS1, SOCS2, SOCS3, SOCS4, SOCS5, SOCS6, and SOCS7) and cytokine-inducible Src homology 2 (SH2) domain-containing protein (CIS, also known as CISH) interact with Cul5 through its “Cul5 box” [51–53]. The amino acid sequence LPΦP (Φ represents a hydrophobic residue) in the Cul5 box is required for specific interaction with Cul5 [51, 53, 54]. Cul5 also interacts with Rbx2, enabling SOCS box-containing proteins to form a protein complex with Cul5 and Rbx2 (Fig. 2) [51, 53, 54] (Table 1).

**Table 1:** Cul5-containing ubiquitin ligases and the corresponding substrates

Cul5-type ubiquitin ligases	Substrates	References
SOCS1	JAK2	[63]
	Vav	[61]
	IRS1 and IRS2	[66]
	GM-CSF receptor βc subunit	[60]
	Cdh1	[65]
	p65	[67]
	Mal	[64]
	HPV E7	[62]
SOCS6	Cas	[69]
SOCS7	Dab1	[86]
SPSB1, 2, and 4	iNOS	[93, 94, 98, 99]
SPSB1	TGF-β type II receptor	[102]
ASB2	Filamin A and B	[107–110]
	Jak3	[112, 113]
ASB3	TNF-R2	[115]
ASB4	IRS4	[118]
	ID2	[124]
ASB6	APS	[125]
ASB9	CKB	[130, 131]
	uMtCK	[132]
ASB11	DeltaA (in *Danio rerio*)	[139, 140]
	Ribophorin 1	[104]
WSB1	HIPK2	[144]
	D2	[160]
	pVHL	[165]
	RhoGDI2	[166]
Rab40	Rap2 GTPase	[167]
Elongin A	Rpb1	[170]
Vif (human immunodeficiency virus)	APOBEC3F	[188]
	APOBEC3G	[186]
BZLF1 (Epstein–Barr virus)	p53	[224, 225]
E1B55K (adenovirus)	p53	[235, 236, 239]
	Mre11	[227]
	DNA ligase IV	[241, 242]
	integrin α3	[243]
	Rep52 and capsid proteins	[244, 245]
LANA (Kaposi’s sarcoma–associated herpesvirus)	pVHL and p53	[254]

**Fig. 3:**
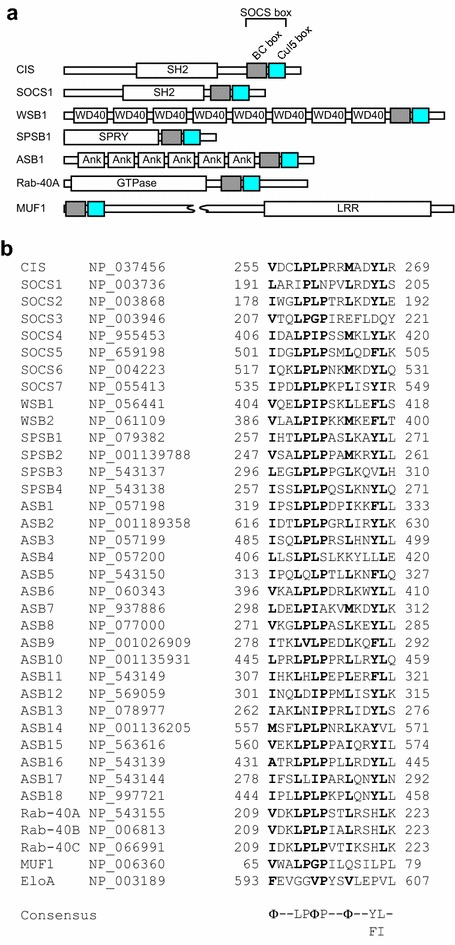
Domain organization of SOCS box proteins. **a** The SOCS box consists of a BC box and a Cul5 box in the order indicated. *SH2* Src homology 2 phosphotyrosine-binding domain, *WD40* WD40 repeats, *SPRY* sp1A/ryanodine receptor domain, *Ank* ankyrin repeats, *LRR* leucine-rich repeats, *GTPase* GTPase domain. **b** Alignment of amino acid sequences of Cul5 boxes present in selected SOCS box proteins. Consensus amino acids are highlighted by *bold font*. The GenBank™ accession numbers of each protein are indicated. *Φ* hydrophobic residue

All CIS/SOCS family proteins have a central SH2 domain and a C-terminally located SOCS box, which consists of an Elongin C-interacting BC box and a Cul5-interacting Cul5 box with an approximately 40-amino acid motif (Fig. 3) [51–58]. CIS/SOCS family proteins bind to janus kinases (JAKs), certain cytokine receptors, or signaling molecules to suppress downstream signaling events [52, 56, 59]. A small kinase inhibitory region (KIR) of SOCS1 and SOCS3 inhibits JAKs by acting as a pseudo-substrate, thereby suppressing further signal transduction [52, 56]. By contrast, CIS/SOCS family proteins inhibit signaling by competing with downstream proteins for binding to the activated receptors, suppressing signal transduction by inducing the polyubiquitination and proteasomal degradation of target substrates [52, 56]. For example, SOCS1 polyubiquitinates JAK2, Vav, IRS1 and IRS2, the GM-CSF receptor βc subunit, Cdh1, p65, Mal, and HPV E7 [60–67].

SOCS1 contains an incompletely conserved Cul5 box, and no interaction between SOCS1 and Cul5 has been detected [51]. Given that SOCS1 polyubiquitinates several substrates as described above, it is possible that the interaction of SOCS1 with these substrates recruits other ubiquitin ligase(s) that actually mediate their polyubiquitination and degradation, or that the bond between SOCS1 and the Cul5/Rbx2 complex is unstable [51]. SOCS1 and SOCS3 bind relatively weakly to Cul5, with affinities 100-fold and 10-fold lower, respectively, than those to the rest of the family [68]. This might explain why only SOCS1 and SOCS3 suppress signal transduction through both SOCS box-dependent and -independent mechanisms [68].

Knockdown of Cul5 accelerates growth factor-independent cell growth, migration, membrane dynamics, and colony dysmorphogenesis, which are all dependent on the endogenous tyrosine kinase Src [69]. Mechanistically, Cul5 and Src stimulate the degradation of the Src substrate p130Cas (Crk-associated substrate) [69]. Tyrosine phosphorylation of Cas stimulates the interaction between SOCS6 and Cas and the proteasomal degradation of Cas [69]. Cas is necessary for the transformation of Cul5 knockdown cells, and Cul5 suppresses epithelial cell transformation by regulating several pathways, including inhibition of Src–Cas-induced ruffling through SOCS6 [69].

Src is a non receptor tyrosine kinase that mediates many signaling pathways involving various soluble and adhesive signaling molecules and regulates cell proliferation, survival, differentiation, and migration [70]. Cul5 downregulates active but not inactive Src, and knockdown of Cul5 increases protein tyrosine phosphorylation, induces morphological transformation, and deregulates cell growth [71].

The mammalian cortical plate assembles from the inside outwards [72, 73]. This organization requires a signaling pathway mediated by an extracellular protein, reelin (Reln), and an intracellular molecule, disabled-1 (Dab1) [74–77]. Reln stimulates the tyrosine phosphorylation of Dab1 by the Src family tyrosine kinases (SFKs) Fyn and Src [78–82]. Tyrosine-phosphorylated Dab1 is degraded in a Cul5 and SOCS protein-dependent manner [83–85]. Functionally, knockdown of Cul5 in migrating neurons shifts their location to a more superficial position, suggesting that Cul5 is crucial for the precise location of the termination of neuronal migration [83]. Furthermore, Rbx2 knockdown resulted in a shift in neuronal positioning to a more superficial location [86]. *Rbx2* conditional knockout mice show neocortical and cerebellar ectopias dependent on Dab1 [86]. Finally, SOCS7 is a Dab1 recognition protein that promotes polyubiquitination and degradation [86].

Tuberous sclerosis complex (TSC) is associated with neurodevelopmental abnormalities resulting from mutations in one of two genes, *TSC1* (encoding hamartin) or *TSC2* (encoding tuberin) [87]. Cul5 is upregulated at the mRNA and protein levels by increased mammalian target of rapamycin (mTOR) signaling or in the absence of Tsc2, providing potential molecular mechanisms underlying the neuronal migration deficit induced by the degradation of Dab1 in TSC pathology [88].

### SPRY domain-containing SOCS box protein (SPSB/SSB) complex

The SplA/ryanodine receptor (SPRY)/B30.2 domain has a role in protein–protein interactions, although its main functions remain poorly understood [89]. The SPRY/B30.2 domain is a sequence repeat in the dual specificity kinase SplA and ryanodine receptors [89].

The four members of the SPSB family (SPSB1–SPSB4) are characterized by a C-terminal SOCS box and a central SPRY/B30.2 domain [89–92]. SPSB1, 2, and 4 polyubiquitinate inducible nitric oxide synthase (iNOS/NOS2), targeting it for proteasomal degradation [93, 94]. The activity of iNOS is approximately tenfold greater than that of NOS1 and NOS3, suggesting that iNOS is a high-output NOS compared with NOS1 and NOS3 [95]. iNOS is not detectable under normal conditions, whereas it is induced in response to cytokines, microbes, or microbial products, resulting in the sustained production of NO [95]. As a result, reactive nitrogen intermediates (such as NO, nitrite, and nitrate) and the products of the interaction of NO with reactive oxygen species (such as peroxynitrite and peroxynitrous acid) accumulate and inhibit viruses or bacteria [95–97]. SPSB2-deficient macrophages show prolonged iNOS and NO production, resulting in the enhanced killing of *L. major* parasites [93]. By contrast, SPSB1 and SPSB4 are major ubiquitin ligases for iNOS that prevent the overproduction of NO, which could cause cytotoxicity [94, 98, 99].

The transforming growth factor-β (TGF-β) signaling pathway is a crucial signaling pathway that requires tight regulation, and dysregulation of this pathway strongly correlates with the progression of human cancers [100, 101]. SPSB1 negatively regulates the TGF-β signaling pathway by ubiquitinating and targeting TGF-β type II receptor (TβRII) for proteasomal degradation [102]. Knockdown of SPSB1 results in the accumulation of TβRII and enhanced TGF-β signaling, migration, and invasion of tumor cells [102].

### Ankyrin repeat and SOCS box (ASB) family

The ASB family is composed of 18 members from ASB1 to ASB18. Several members interact with Cul5-Rbx2 and act as ubiquitin ligase complexes [103]. ASB-Cul5 complexes can oligomerize, and Cul5 can form heterodimeric complexes with the Cul4a-DDB1 complex [104].

Although ASB1 is expressed in multiple organs, including the hematopoietic compartment, ASB1-deficient mice develop normally and exhibit no phenotypes, with the exception of diminished spermatogenesis and incomplete filling of seminiferous tubules [105].

ASB2 is induced by retinoic acid (RA) in acute promyelocytic leukemia cells, and exogenous ASB-2 in myeloid leukemia cells results in growth inhibition and chromatin condensation, which recapitulate the early steps of induced differentiation of acute promyelocytic leukemia cells [106]. ASB2 targets the actin-binding proteins filamin A and B for proteasomal degradation [107–110]. Knockdown of ASB2 in leukemia cells delays RA-induced differentiation, which suggests that ASB2 regulates hematopoietic cell differentiation by targeting filamins for degradation, thereby modulating actin remodeling [107]. ASB2 enhances the adhesion of hematopoietic cells to fibronectin, the main ligand of β1 integrins, by promoting filamin A degradation [111]. ASB2 heterodimerizes with Skp2 and forms a noncanonical Cul1- and Cul5-containing dimeric ubiquitin ligase complex that promotes the polyubiquitination and degradation of Jak3 [112, 113]. A list of candidate substrates of ASB2 was reported in a recent study [114].

Tumor necrosis factor receptor type 2 (TNF-R2) is polyubiquitinated by ASB3 and targeted for proteasomal degradation [115]. Thereby, ASB3 negatively regulates TNF-R2-mediated cellular responses initiated by TNF-α [115].

Insulin receptor substrate 4 (IRS4) is expressed predominantly in the pituitary, thymus, and brain [116]. IRS4 is an adaptor molecule involved in signal transduction by both insulin and leptin, and is widely expressed throughout the hypothalamus [117]. ASB4 colocalizes and interacts with IRS4 in hypothalamic neurons and polyubiquitinates IRS4 for degradation to decrease insulin signaling [118]. Downregulation of ASB4 in HCC cells hinders cell migration and invasion, whereas overexpression of ASB4 increases the migration rate; ASB4 is downregulated by miR-200a [119]. ASB4, which is highly differentially expressed in the vascular lineage during development [120], is an oxygen-sensitive ubiquitin ligase that is abundantly expressed in the developing placenta and is upregulated during the differentiation of embryonic stem cells into endothelial cell lineages [121]. Inhibitor of DNA binding 2 (ID2) negatively regulates vascular differentiation during development [122, 123], and ASB4 promotes the ubiquitination and proteasomal degradation of ID2 [124]. ASB4-deficient mice phenocopy human pre-eclampsia, including hypertension and proteinuria in late-stage pregnant females, indicating that ASB4 mediates vascular differentiation in the placenta through the degradation of ID2 [124].

ASB6 is expressed in 3T3-L1 adipocytes but not in fibroblasts, and may regulate the insulin signaling pathway in adipocytes by promoting the degradation of adapter protein with a pleckstrin homology and SH2 domain (APS) [125].

The crystal structure of ASB9 with or without Elongin B and C has been determined [126–128]. ASB9 alone is unstable, whereas it forms a stable complex with Elongin B and C that also binds with high affinity to the Cul5N-terminal domain (Cul5NTD) but not to Cul2NTD [129]. ASB9 polyubiquitinates and decreases the levels of creatine kinase B (CKB) and ubiquitous mitochondrial creatine kinase (uMtCK) [130–132]. CK plays a major role in cellular energy metabolism in non-muscle cells [133]. CKB is overexpressed in a number of tumors, including neuroblastoma, small cell lung carcinoma, colon and rectal adenocarcinoma, and breast and prostate carcinoma [133, 134]. Furthermore, high ASB9 mRNA expression is correlated with good prognosis, and knockdown of ASB9 increases colorectal cancer (CRC) cell invasiveness [135]. ASB9 upregulation may result in a good prognosis for CRC by promoting the degradation of CKB and uMtCK.

The Notch signaling pathway is essential for the spatio-temporal regulation of cell fate [136–138]. The single-pass transmembrane protein delta acts as a ligand for the Notch receptor. *Danio rerio* Asb11 (d-Asb11) regulates compartment size in the endodermal and neuronal lineages by promoting the ubiquitination and degradation of deltaA but not deltaD, leading to the activation of the canonical Notch pathway [139, 140]. Knockdown of d-Asb11 downregulates specific delta-Notch elements and their transcriptional targets, whereas these are induced when d-Asb11 is misexpressed in zebrafish embryos [139]. These data indicate that d-Asb11 regulates delta- Notch signaling for the fine-tuning of lateral inhibition gradients between deltaA and Notch [139]. Mutant zebrafish lacking the Cul5 box, which results in the inability to degrade delta, are defective in Notch signaling, as indicated by the impaired expression of Notch target genes [141].

Forced expression of d-asb11 impairs terminal differentiation and increases proliferation in the myogenic progenitor compartment [142]. By contrast, mutation of d-asb11 causes premature differentiation of muscle progenitors and delays regenerative responses in adult injured muscle, suggesting that d-asb11 is a principal regulator of embryonic as well as adult regenerative myogenesis [142]. ASB11 is an endoplasmic reticulum (ER)-associated ubiquitin ligase that promotes the ubiquitination and degradation of Ribophorin 1, an integral protein of the oligosaccharyltransferase (OST) glycosylation complex, which *N*-glycosylates newly synthesized proteins in the rough ER [104, 143].

### WD repeat and SOCS box-containing protein 1 (WSB1)

WSB1 polyubiquitinates homeodomain-interacting protein kinase 2 (HIPK2) [144]. HIPK2 interacts with a variety of transcription factors, the p300/CBP co-activator, and the Groucho/TLE co-repressor [145–152]. Functionally, HIPK2 prevents apoptosis mediated by p53, CtBP, Axin, Brn3, Sp100, TP53INP1, and PML [153–157]. The loss of HIPK2 reduces apoptosis and increases the numbers of trigeminal ganglia, whereas overexpression of HIPK2 in the developing sensory and sympathetic neurons promotes apoptosis [153, 158]. DNA damaging agents such as adriamycin or cisplatin prevent the WSB1-mediated degradation of HIPK2, which thereby remains active and stable for the induction of apoptosis [144].

WSB1 is induced by sonic hedgehog (Shh) in developing limb buds and other embryonic structures [159]. Thyroid hormone-activating enzyme type 2 iodothyronine deiodinase (D2) is polyubiquitinated by WSB1 [160]. Ubiquitination of Shh-induced D2 by WSB1 induces parathyroid hormone-related peptide (PTHrP), thereby regulating chondrocyte differentiation [160].

Although WSB1 binds to the interleukin-21 receptor (IL-21R), WSB1 inhibits the degradation of the mature form of IL-21R [161]. Mechanistically, WSB1 associates with the intracytoplasmic region of IL-21R and facilitates the maturation of IL-21R from an N-linked glycosylated form to a fully glycosylated mature form [161].

The von Hippel-Lindau tumor suppressor pVHL is a ubiquitin ligase that targets hypoxia-inducible factor-α (HIF-α) for proteasomal degradation in normoxia [162, 163]. Dysregulation and accumulation of HIF-α upregulates downstream target gene expression and contributes to tumor progression, promoting invasion, metastasis, and angiogenesis [162, 163]. WSB1 is induced under hypoxic conditions [164] and promotes pVHL ubiquitination and proteasomal degradation, thereby stabilizing HIF-α under both normoxic and hypoxic conditions [165]. WSB1 upregulates gene expression regulated by HIF-1α and promotes cancer invasion and metastasis [165]. In a recent study, quantitative proteomic screening and functional analyses revealed that WSB1 promotes the ubiquitination and proteasomal degradation of the Rho-binding protein RhoGDI2, thereby activating Rac1 to stimulate tumor cell motility and invasion in hypoxia-driven osteosarcoma [166].

### Rab40 complex

Xenopus homolog of Rab40 (XRab40) is localized at the Golgi apparatus and interacts with Elongin B/C and Cul5 [167]. Although the XRab40 complex ubiquitinates the Rap2 GTPase, it may not destabilize Rap2 [167]. The XRab40 complex regulates the membrane localization of dishevelled (Dsh), a key signaling molecule in the Wnt pathway, through Rap2 and its effector misshapen/Nck-interacting kinase (XMINK) [167]. The XRab40 complex, Rap2, and XMINK are suggested to play a crucial role in the regulation of the noncanonical Wnt pathway.

### MUF1 complex

MUF1 binds the Cul5/Elongin BC complex and has ubiquitin ligase activity; however, its substrate has not been identified to date [168]. MUF1 is a ubiquitously expressed nuclear protein that, upon coexpression with RhoBTB, a Cul3-type ubiquitin ligase, is partially retained in the cytoplasm, where both proteins colocalize [169].

### Elongin ABC complex

The Elongin ABC complex interacts with Cul5 and Rbx2 and polyubiquitinates the large subunit of RNA polymerase II (Rpb1) in response to UV irradiation [170].

UV irradiation leads to the phosphorylation of Rpb1 at Ser5, which increases the interaction between Elongin A and Rpb1 [170]. UV irradiation-dependent ubiquitination and proteasomal degradation of Rpb1 are significantly suppressed in Elongin A-deficient cells [170].

## Virus-related Cul5-containing ubiquitin ligases

### Human immunodeficiency virus-1 (HIV-1)

**Fig. 4:**
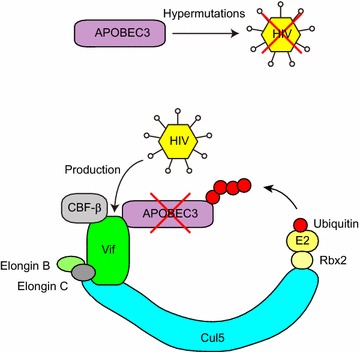
Degradation of APOBEC3 by the HIV Vif protein. APOBEC3 introduces nonsense and/or missense mutations in the HIV genome, thereby showing antivirus activity. The HIV-1 Vif protein forms a complex with Cul5, the Elongin B/C heterodimer, Rbx2, E2, ubiquitin (Ub), and CBF-β. The Vif complex targets APOBEC3 for polyubiquitination and proteasomal degradation

Apolipoprotein B editing complex 3G (CEM15/APOBEC3G)(A3G), a human cytidine deaminase, is a broad antiviral factor against human HIV-1, simian immunodeficiency virus (SIV), mouse leukemia virus, and hepatitis B virus [171–179]. A3G induces C to U mutations in the viral minus DNA strand during reverse transcription, resulting in deleterious G to A mutations in the coding strand (Fig. 4) [171, 173–175, 179–181].

The HIV-1 virion infectivity factor (Vif) is essential for viral evasion of the host antiviral factor A3G [182, 183]. Vif interacts with Cul5, Elongins B and C, and Rbx1/Rbx2 [184–186]. This complex interacts with A3G and induces its ubiquitination and degradation (Fig. 4) [185–187]. HIV Vif can also bind APOBEC3F (A3F) and induce its polyubiquitination and degradation [188]. The SIV from rhesus macaques (SIVmac) Vif also forms a Cul5-containing ubiquitin ligase complex in human cells [186], and neddylation of Cul5 by the NEDD8-conjugating enzyme UBE2F is required for Vif-mediated degradation of A3G [189].

In the absence of the Vif protein, A3G is packaged into viral particles and functions by hypermutating viral DNA in the newly infected cell [171, 173–176, 179]. Lysine-free A3G (all lysine residues are mutated to arginine) is still degraded by the proteasome in a Vif-dependent manner [190], and polyubiquitination of Vif is critical for A3G proteasomal degradation [190].

Infection with HIV-1 causes cell cycle arrest or delay in the G2 phase, when the expression of the viral genome is optimal and long terminal repeat (LTR) is most active [191–193]. Several controversial reports suggest that viral protein R (Vpr) and/or Vif mediate cell cycle arrest. Vpr of HIV-1 alter the cell cycle by inhibiting the activation of Cdc2/Cdk1, a G2/M checkpoint regulating kinase, to prevent or delay entry into mitosis [194–196]. Vif and Vpr acting together, but not alone, cause G2 arrest [197]. However, Vif was reported to cause G2 arrest [198], and also to block Vpr-mediated G2 arrest [199]. Nevertheless, Vif-mediated G2 arrest is Cul5-dependent [200]. Vif also recruits the transcription cofactor CBF-β, which is required for Vif-mediated degradation of A3G but not A3A [201–203]. CBF-β is a subunit of a heterodimeric transcription factor without DNA-binding activity that regulates the folding and DNA-binding activity of partner RUNX family proteins, which is crucial for the development and differentiation of diverse cell types, including T lymphocytes [203–205].

Vif is phosphorylated on several serine and threonine residues, among which Ser144 plays a crucial role in regulating HIV-1 replication [206, 207]. Mutation of Ser144 to Ala suppresses Vif activity and causes >90 % inhibition of HIV-1 replication [206]. Mechanistically, phosphorylation at Ser144 negatively regulates the binding of the Vif BC box to Elongin C [208].

Vif contains a BC box and a SOCS box that are required for the interaction with ElonginB/C and Cul5, respectively [51, 209, 210]. Binding of Elongin B/C changes the conformation of Vif, facilitating its interaction with CBF-β and Cul5 [211]. Although both Rbx1 and Rbx2 can interact with Cul5, only the knockdown of Rbx2, but not that of Rbx1, impairs Vif-induced A3G degradation [212].

Susceptibility to HIV-1 and disease progression may be affected by variation in human genes [213, 214]. Cul5 is one of the genes in which signatures of selection have been reported [215]. Several single nucleotide polymorphisms (SNPs) in the CUL5 locus have been identified and shown to affect the rate of CD4^+^ T cell loss in patients infected with HIV-1 [216]. Cul5 haplotypes are grouped into two clusters with opposing effects, as cluster I delays and cluster II accelerates CD4^+^ T cell loss [216]. Reduced APOBEC3 activity is associated with the Cul5 SNP6 minor allele [217]; however, the Cul5 SNP6 has no effect on vertical transmission or progression to pediatric AIDS [218].

### Epstein–Barr virus (EBV)

EBV, a human γ-herpesvirus, is associated with several B cell and epithelial cell malignancies, and there are two different infection states, latent and lytic [219]. BZLF1 (known as Zta, EB1, or ZEBRA) is a transcriptional transactivator that induces EBV early gene expression to promote an EBV lytic cycle cascade [220–223]. BZLF1 contains both a Cul2 box and a Cul5 box, thereby binding to both Cul2 and Cul5 [224]. BZLF1 polyubiquitinates and induces the degradation of p53, which inhibits apoptosis and is required for efficient viral propagation in the lytic replication stage [224, 225].

### Human adenoviruses (Ad)

**Fig. 5:**
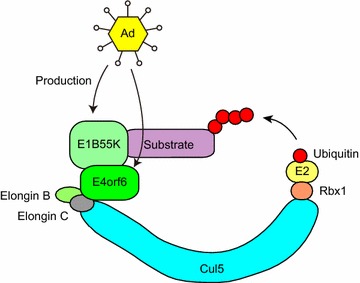
Degradation of substrate proteins by the adenoviral proteins E1B55K and E4orf6. The adenoviral protein E1B55K recognizes substrates to be polyubiquitinated, and also interacts with another adenoviral protein, E4orf6. E4orf6 further interacts with the Elongin B/C heterodimer, Cul5, and Rbx1, E2, and ubiquitin (Ub)

Human Ad are classified into six groups (A–F), and they comprise a large family of more than 50 different serotypes [226]. The human adenovirus type 5 (Ad5) early-region 4 34 kDa product from open reading frame 6 (E4orf6) contains three BC boxes [227–229]. Although Ad5 E4orf6 forms a complex containing Cul5, Elongin B, Elongin C, and Rbx1, a Cul5 box is not found in Ad5 E4orf6 (Fig. 5) [227, 229, 230]. Adenoviral early-region 1B 55 kDa protein (E1B55K) associates with E4orf6 and the complex targets substrates for proteasomal degradation [227, 228, 231]. Although efficient substrate degradation is dependent on the interaction with E1B55K in some cases, several substrates efficiently bind to E1B55K but are not degraded, whereas others are degraded without detectable interactions with E1B55K [232]. These results indicate that transient interactions with E1B55K may be sufficient for substrate degradation and that the orientation of the substrate in the ubiquitin ligase complex is probably crucial [232].

The E4orf6/E1B55K complex is essential for efficient viral replication, and some of its key substrates have been identified, such as p53 [233–239], meiotic recombination 11 (Mre11) [227, 240], DNA ligase IV [241, 242], integrin α3 [243], and the adeno-associated virus type 5 (AAV5) Rep52 and capsid proteins [244, 245].

The Mre11 complex, which consists of Mre11, RAD50, and Nijmegen breakage syndrome 1 (NBS1, also known as nibrin), detects DNA double strand breaks (DSBs) and induces p53-dependent apoptosis [246]. DNA ligase IV plays a pivotal role in repairing DSBs, and the mutation of this gene results in ligase IV (LIG4) syndrome, which is characterized by pronounced radiosensitivity, genome instability, malignancy, immunodeficiency, and bone marrow abnormalities [247]. The heterodimer of integrin α and β subunits functions as a transmembrane receptor that links external signals to intracellular signaling pathways. For example, integrin α3β1 binds a variety of extracellular matrix substrates, including fibronectin, collagen, vitronectin, and laminins [248]. Degradation of integrin α3 mediated by the E4orf6/E1B55K complex might be involved in cell detachment from the extracellular matrix, which may contribute to virus spread [243].

Although the human Ad5 E4orf6 complex binds Cul5, Cul2 is primarily present in the Ad12 and Ad40 E4orf6 complexes, as they contain a Cul2 box [229, 249]. The Ad16 E4orf6 complex binds Cul2 as well as Cul5 and is not able to degrade p53 and integrin α3 [229].

The anti-apoptotic protein Gam1 is an essential viral protein encoded by the avian adenovirus CELO (chicken embryo lethal orphan) [250, 251] that inhibits cellular sumoylation [252]. Gam1 contains a SOCS box-like domain and binds Cul2, Cul5, Elongin B/C, and Rbx1, targeting the SUMO E1 enzyme SAE1 for polyubiquitination and degradation [253].

### LANA complex

Kaposi’s sarcoma-associated herpesvirus (KSHV)-encoded latency-associated nuclear antigen (LANA) contains a putative SOCS box and forms a complex with Elongin B/C and Cul5 [254]. This complex promotes the polyubiquitination and degradation of pVHL and p53 [254, 255]. Thus, LANA provides a favorable environment for the progression of KSHV-infected tumor cells by downregulating tumor suppressors.

## Substrates of Cul5 (adaptor protein is unknown)

### DEPTOR

DEPTOR binds mTOR and inhibits the mTOR complex 1 (mTORC1) and mTORC2 pathways [256]. DEPTOR accumulates upon nutrient deprivation and contributes to the induction of autophagy. In response to mitogens, DEPTOR is phosphorylated on three serine residues in a conserved degron and is recognized by F box protein βTrCP for polyubiquitination and consequent proteasomal degradation [257–259]. The Cul5/Elongin B complex also targets DEPTOR for ubiquitin-proteasomal degradation under nutrient-rich conditions, and knockdown of Cul5, but not of Cul2, results in autophagy induction [260]. Thus, Cul5 temporally controls the autophagy response.

### Heat shock protein 90 (Hsp90) client proteins

Hsp90 is a molecular chaperone that facilitates the stabilization and activation of approximately 350 client proteins [261]. Pharmacologic inhibition of Hsp90 results in the Cul5 and Rbx2-dependent proteasomal degradation of client proteins including ErbB2, BRAF(V600E), AKT, CDK4, and HIF-1α, indicating the crucial role of Cul5 in the response to Hsp90 inactivation [262–266]. ErbB2 degradation mediated by Cul5 is independent of Elongin B/C function, as indicated by the fact that dominant negative Elongin C, which can bind Cul5 but not the SOCS box in the substrate receptor, has no effect on the degradation of ErbB2 [262].

### TRIAD1

Two RING fingers and DRIL (double RING finger linked) 1 (TRIAD1) contains a RING-in-between-RING (RBR) domain and markedly inhibits myeloid colony formation [267]. TRIAD1-deficient mice die because of a severe multiorgan immune response [268]. Binding of neddylated Cul5 and Rbx2 to TRIAD1 enhances TRIAD1 ubiquitin ligase activity [269].

## Conclusions

Cul5-containing ubiquitin ligases regulate a variety of signaling pathways by targeting particular substrates for proteasomal degradation or competing for protein–protein interactions. However, many Cul5-containing ubiquitin ligases remain to be studied, and a complete list of substrates or binding proteins of Cul5 is not available. Considering that some viruses hijack Cul5 to degrade antiviral proteins, it might be better to study the function of Cul5 during virus infection. Certain viruses target Elongin C-interacting Cul5 (and in some cases Cul2) for hijacking, although the cause remains undetermined. Studies focusing on Elongin C might shed light on the physiological functions of Cul5.

## Abbreviations

Ad: adenoviruses
APS: adapter protein with a pleckstrin homology and SH2 domain
AQP: aquaporin
ASB: ankyrin repeat and SOCS box
AVP: arginine vasopressin
A3F: apolipoprotein B editing complex 3F
A3G: apolipoprotein B editing complex 3G
Cas: Crk-associated substrate
CIS: cytokine-inducible Src homology 2 (SH2) domain-containing protein
CKB: creatine kinase B
Dab1: disabled-1
Dsh: dishevelled
D2: thyroid hormone-activating enzyme type 2 iodothyronine deiodinase
EBV: Epstein–Barr virus
EGR-1: early growth response 1
E1B55K: early-region 1B 55 kDa protein
E4orf6: early-region 4 34 kDa product from open reading frame 6
HIF: hypoxia-inducible factor
HIPK2: homeodomain-interacting protein kinase 2
HIV-1: human immunodeficiency virus-1
ID2: inhibitor of DNA binding 2
IL: interleukin
iNOS: inducible nitric oxide synthase
IRS: insulin receptor substrate
JAKs: janus kinases
KIR: kinase inhibitory region
LANA: latency-associated nuclear antigen
MAPK: mitogen activated protein kinase
miR: microRNA
NO: nitric oxide
PKA: protein kinase A
pVHL: von Hippel-Lindau tumor suppressor
RA: retinoic acid
RAMECs: rat adrenal medullary endothelial cells
Reln: reelin
SFKs: Src family tyrosine kinases
Shh: sonic hedgehog
SH2: Src homology 2
SIV: simian immunodeficiency virus
SIVmac: SIV from rhesus macaques
SOCS: suppressor of cytokine signaling
SPRY: SplA/ryanodine receptor
SPSB: SPRY domain-containing SOCS box
TBI: traumatic brain injury
TGF-β: transforming growth factor-β
TNF: tumor necrosis factor
TRIAD1: two RING fingers and DRIL (double RING finger linked) 1
TSC: tuberous sclerosis complex
VACM: vasopressin-activated calcium-mobilizing
Vif: virion infectivity factor
Vpr: viral protein R
WSB1: WD repeat and SOCS box-containing protein 1
XMINK: Xenopus misshapen/Nck-interacting kinase
